# HIV vulnerability among adolescent girls and young women: a multi-country latent class analysis approach

**DOI:** 10.1007/s00038-020-01350-1

**Published:** 2020-04-09

**Authors:** Sanyukta Mathur, Nanlesta Pilgrim, Sangram Kishor Patel, Jerry Okal, Victor Mwapasa, Effie Chipeta, Maurice Musheke, Bidhubhusan Mahapatra, Julie Pulerwitz

**Affiliations:** 1 Population Council, 4301 Connecticut Ave, Suite 280, Washington, DC USA; 2 Independent Consultant, Washington, DC USA; 3 Population Council, New Delhi, India; 4 Population Council, Nairobi, Kenya; 5 Centre for Reproductive Health, College of Medicine, Blantyre, Malawi; 6 Independent Consultant, Lusaka, Zambia

**Keywords:** Transactional sex, IPV, Gender equity, STI, Condom use

## Abstract

**Objectives:**

To stem the HIV epidemic among adolescent girls and young women (AGYW, 15–24 years), prevention programs need to reach AGYW who are most at risk. We examine whether individual- and household-level factors could be used to define HIV vulnerability for AGYW.

**Methods:**

We surveyed out-of-school AGYW in urban and peri-urban Kenya (*N* = 1014), in urban Zambia (*N* = 846), and in rural Malawi (*N* = 1654) from October 2016 to 2017. LCA identified classes based on respondent characteristics, attitudes and knowledge, and household characteristics. Multilevel regressions examined associations between class membership and HIV-related health outcomes.

**Results:**

We identified two latent classes—high and low HIV vulnerability profiles—among AGYW in each country; 32% of the sample in Kenya, 53% in Malawi, and 51% in Zambia belonged to the high vulnerability group. As compared to AGYW with a low-vulnerability profile, AGYW with a high-vulnerability profile had significantly higher odds of HIV-related outcomes (e.g., very early sexual debut, transactional sex, sexual violence from partners).

**Conclusions:**

Out-of-school AGYW had differential vulnerability to HIV. Interventions should focus on reaching AGYW in the high HIV vulnerability profiles.

## Introduction

Nearly four decades into the HIV epidemic, HIV rates among adolescent girls and young women (AGYW, females aged 15–24 years) remain intractable in many settings. Over 1000 AGYW become infected with HIV daily (UNAIDS 2016b). In eastern and southern Africa, AGYW are at considerably higher risk of HIV acquisition compared to their male counterparts. According to estimates, of the nearly 290,000 new HIV infections in eastern and southern Africa among 15- to 24-year olds, two-thirds occurred among AGYW, and HIV and AIDS remain the leading cause of death among AGYW in this region (UNAIDS 2016a).

AGYW vulnerability to HIV is multifaceted—shaped by a range of proximal biological and behavioral factors, as well as more distal social and structural factors like gender norms (Harrison et al. 2015; Santelli et al. 2015). For instance, HIV incidence is higher when young women also have sexually transmitted infections (STIs) (Santelli et al. 2013). Behaviorally, engaging in transactional sex, having multiple partnerships, engaging in substance abuse, and limited condom use also contribute to HIV risk among AGYW (Jewkes et al. 2010; Santelli et al. 2013). Further, structural factors, like parental loss and being out-of-school, are associated with HIV acquisition (Birdthistle et al. 2008). Prior research in Zambia, for instance, shows that gender inequality and poverty undermine HIV prevention among AGYW (Butts et al. 2017). Yet, each of these factors individually is not consistently associated with HIV acquisition among AGYW in different contexts (Napierala Mavedzenge et al. 2011). For instance, research in Malawi found that consistent condom use was not associated with HIV status among AGYW (Price et al. 2018). This complexity denotes the need to better assess the combination of factors that contribute to HIV risk among AGYW considering the multi-dimensionality of HIV risk among AGYW.

To stem the HIV epidemic among AGYW, HIV prevention program efforts need to reach AGYW who are most at risk of HIV acquisition. However, while AGYW’s disproportionate risk is widely recognized, it is less clear which AGYW are most at risk (Price et al. 2018). The variability in risk factors across different contexts and the uneven distribution of risk creates confusion around how best to define HIV vulnerability for AGYW as well as challenges around whom to recruit into programs (Underwood et al. 2009). The current approach of segmenting AGYW by socio-demographic factors, like age and marital status, is often insufficient for differentiating HIV risk or vulnerability groups. For instance, targeting by marital status is common, yet research shows that it is not necessarily a risk factor for HIV acquisition. It also remains unclear whether the focus should be on individual-level behavioral factors, household-level characteristics, or broader structural factors in defining HIV vulnerability. Generating HIV vulnerability profiles based on factors that synergistically affect HIV and related outcomes among AGYW could be used by programmers for more effective targeting and assessment of HIV prevention efforts (Edelstein et al. 2013; Population Council 2015; Underwood and Schwandt 2015). However, there is limited utilization of methods in sub-Saharan Africa that could assess underlying groupings of risk factors to develop segmented HIV vulnerability profiles of AGYW.

Another challenge faced by current efforts during program-level screening for HIV vulnerability among AGYW in a community is the potential to exacerbate stigma and discrimination (Denison et al. 2017). Going beyond socio-demographic segmentation, some efforts to reach the most at-risk young women are based on their sexual practices. A recent mapping of risk assessment tools to help identify/enroll individuals at substantial risk of HIV infection found that key criteria for AGYW included socio-demographic characteristics (age of AGYW, age of sexual debut, age of partner), sexual behaviors (number of partners, condom use, transactional sex), and relationship characteristics (primary partner’s HIV status or use of antiretroviral drugs), and exposure to violence (Dunbar et al. 2018). However, asking young women about sensitive topics like sexual activity, number of partners, coital frequency, and condom use in community settings where adolescent sexuality or HIV may already be stigmatized can subject young women to greater scrutiny and potentially lead to stigma and discrimination toward them. It may also deter young women from engaging in HIV prevention programs. Thus, the challenge remains to utilize information from non-sensitive questions to better identify HIV vulnerability.

This analysis explores whether underlying factors that influence proximate determinants of HIV risk (e.g., risky sexual behavior) could be used to define HIV vulnerability for AGYW (Boerma and Weir 2005). Using the proximate determinants theoretical framework, which highlights how underlying determinants can influence proximate determinants of HIV risk, we examine whether household characteristics and respondent characteristics, attitudes, and knowledge can be used to effectively define HIV vulnerability among AGYW (Boerma and Weir 2005; Santelli et al. 2015). In this paper, we provide empirically developed HIV vulnerability profiles for AGYW in three country contexts—Kenya, Malawi, and Zambia. We use latent class analysis (LCA), a mixed analytical model that aims to uncover unobserved heterogeneity in a population and to find substantively meaningful groups of people that are similar in their responses to measured variables (Muthén 2001, 2004). We use a range of low-sensitivity factors/underlying determinants (that may be less likely to bring scrutiny and stigma toward AGYW) that may co-occur and reinforce AGYW’s vulnerability and diminish their capacity and agency to enact preventative behaviors or practices. In each context, we assess the associations between the vulnerability profiles and HIV-related health outcomes.

## Methods

### Study population

Cross-sectional survey data were collected with AGYW aged 15–24 years from eight study sites across Kenya, Malawi, and Zambia. In Kenya, the study sites included an urban and a peri-urban community in Kisumu County. In Malawi, the study sites included four rural sites in Zomba and Machinga districts. In Zambia, the study sites included two urban communities, one in the capital city of Lusaka and another in the central region of Ndola. These study sites were part of the U.S. President’s Emergency Plan for AIDS Relief (PEPFAR)-supported DREAMS partnership program, focused on reducing HIV risk and incidence among AGYW and their male partners (Saul et al. 2018). DREAMS program locations were selected by PEPFAR in consultation with local government representatives and other stakeholders in each country. In general, the DREAMS program communities are characterized by high HIV prevalence rates among AGYW. The study sites were purposively selected, in consultation with PEPFAR colleagues and DREAMS implementing partners, to be representative of key geographic characteristics (e.g., urban/rural) of DREAMS program communities in each country.

Eligible survey participants were females aged 15–24 years residing in the study catchment area, who intended to stay in the area for the subsequent year, and agreed to participate in the survey. In Kenya, 1014 out-of-school AGYW were interviewed from October 2016 to February 2017. In Zambia, 846 out-of-school AGYW were interviewed from November 2016 to April 2017. In Malawi, 1653 out-of-school AGYW were interviewed from July 2017 to September 2017. Using the DREAMS program beneficiary rosters (in all three countries) and household listings (in Kenya and Zambia) for the program sites prepared by the program implementing partners, we conducted an age-stratified random sample to select potential respondents. Respondents were randomly sampled from participants who were enrolled in the DREAMS program and other AGYW residing in the catchment area of the study sites. Twenty respondents in Kenya, 33 in Zambia, and 3 in Malawi refused to participate due to lack of parental consent or limited time availability at the time of the interview.

Comprehensive surveys captured information on socio-demographic characteristics, sexual behaviors, partnership characteristics, social assets, and HIV outcomes (e.g., reported HIV status, STI symptoms, and HIV testing). The surveys were administered by trained female interviewers and conducted in a local language of the respondent’s choosing (English, Kiswahili, Luo, and English in Kenya; English, Bemba, or Nyanja in Zambia; and Chichewa and Yao in Malawi). Interviews were conducted in private yet convenient locations to the respondents (e.g., room in respondent’s home, nearby field, or nearby community center), and out of earshot of parents, guardians, or other community members.

### Measures

For the LCA model, we considered four key domains aiming to tap into underlying factors associated with HIV acquisition among AGYW: household characteristics, respondent characteristics, attitudes, and knowledge (Table 1).

**Table 1:** Variables used in the latent class analysis to develop HIV risk profiles for out-of-school 15- to 24-year-old women in Kenya, Malawi, and Zambia, 2016–2017

Domains and factors	Measurement and variable definition
*Household characteristics*	
Orphanhood	Questions asked about loss of mother and father, defined as lost both parents, lost mother, lost father, and non-orphan
Household socioeconomic status	Composite measure created of three questions about household construction, water, and sanitation facilities and divided into tertiles of low, mid, and high
Hunger in the past month	Question about going without food for a whole day in the past month, defined as often/sometimes versus rarely/never
Adult supervision	Questions asked whether an adult in the household knows the respondent’s whereabouts during day or at night, defined as yes versus no
*Respondent characteristics*	
Mobility	Question on movement or travel outside of community, defined as weekly/monthly, yearly, never
Marital status	Question asked about marital status (formal or civil or cohabitation as if married) of the respondents, defined as currently married, formerly married, and never married
*Respondent attitudes*	
Support for gender equitable norms	Attitudinal measures assessed support for gender equitable norms using the previously validated GEM scale. The GEM scale assesses views on dimensions on home and child care (e.g., cooking and cleaning are the wife’s responsibility), sexual relationships (e.g., men are always ready to have sex), health and disease prevention (e.g., my partner would be outraged if I asked him to use a condom), and violence (e.g., there are times when a woman deserves to be beaten). An overall GEM scale score is calculated and categorized as low, medium, and high support for equitable gender norms (Pulerwitz and Barker 2008; Vu et al. 2017; Wesson et al. 2019)
Perception on exposure to HIV	Question on self-perception of HIV risk, “How likely is it that you have been exposed to HIV,” defined as (no risk/unlikely versus somewhat likely/very likely/don’t know) (Santelli et al. 2013)
*Respondent knowledge*	
Comprehensive knowledge about HIV transmission	Composite measure based on standard Demographic Health Survey questions of correct knowledge of two ways to prevent HIV (e.g., can people reduce their chance of getting HIV by having just one uninfected sex partner who has no other sex partners) and rejection of three misconceptions about HIV (e.g., can people get the HIV/AIDS virus from mosquito bites), defined as yes/no
Comprehensive knowledge about condoms	Composite measure based on questions around correct knowledge that condoms are an effective method of preventing pregnancy, protecting against HIV/AIDS, and protecting against STIs, defined as yes/no

Multilevel logistic regression models were used to validate the latent class solution or HIV risk profile, for different outcome variables (Table 2).

**Table 2:** Outcome variables used in the multilevel logistic regression models in Kenya, Malawi, and Zambia

Self-reported HIV status	Self-reported HIV status was asked as “what were the results of your HIV test or the last test from which you received results?” with response options of HIV positive, HIV negative, don’t know, and never tested, defined as HIV positive versus other responses
Experience of STI symptoms	Composite measure that asked about experience with STI symptoms (e.g., painful urination, genital ulcers) in the last 6 months, defined as yes to any symptom versus no to all
Early age at sexual debut	Question on self-reported age at first sex, defined as ≤ 15 years versus ≥ 16 years
Pregnancy experience	Question on any pregnancy experience by the partners, defined as yes versus no
Multiple sexual partners in the last year	Question on the number of sexual partners in the last year, defined as 0/1 versus ≥ 2
Engaged in transactional sex	Composite measure of a set of questions about having sex with any partner in the last 12 months because the respondent expected to receive or received money somewhere to stay, or other material goods such as food or support for children, defined as yes to any versus no to all
Experience of physical violence from intimate partners in the last 12 months	Composite measure from a set of questions that asked if AGYW experienced any of following in the last 12 months: slapped or had something thrown at you which could hurt you; pushed or shoved you; hit you with a fist or something else which could hurt you; kicked, dragged, beaten, choked, or burnt you; and threatened to use or actually used a gun, knife, or other weapon against you, defined as yes to any versus no to all
Experience of sexual violence from intimate partners in the last 12 months	Composite measure from a set of questions about sexual violence from an intimate partner was defined as an AGYW experiencing any of following in the last 12 months: physically forced into sex, threatened into sex, and forced to do any other sexual act versus no to all
Experience of sexual violence from non-partners in the last 12 months	Composite measure asking about AGYW experiencing any of the following in the last 12 months: forced or persuaded you to have sex against will, tried to force you to have sex, forced to have sex while you were too drunk or drugged to refuse, and two or more men forced you to have sex with them at the same time
Condom use at last sex	Question about condom use at last sex with main partners, defined as yes versus no
HIV testing in the last 12 months	Question about HIV testing in the last 12 months, defined as yes versus no

### Analysis

LCA was used for HIV risk vulnerability classification. To decide the number of classes and best fit models, we used Akaike’s information criterion (Akaike 1973, 1987), Bayesian information criterion (BIC) (Schwarz 1978), entropy (Celeux and Soromenho 1996), and the Lo–Mendell–Rubin likelihood ratio test (LMR test) (Lo et al. 2001). The LMR test was used to test the number of classes in this mixture analysis procedure; the former is obtained by running the *k*-class and *k* − 1 class analyses and using the derivatives from both models to compute the *p* value (a low *p* value rejects the *k* − 1 class model in favor of the k-class model) (Asparouhov and Muthén 2012). The classification quality of the model was evaluated according to the entropy criterion, in which the values range from zero to one, where values close to one indicate good classification. LCA was conducted using Mplus software (v6.12).

We examined four-, three-, and two-class models. The four- and three-class models did not fit the data well; thus, we focused analyses on the two-class models. The *p*-values of the LMR test supported the two-class solutions (Kenya: *p* = 0.032; Zambia: *p* = 0.073; Malawi: *p* ≤ 0.0001) as the three-class solutions did not improve the model fit compared to the two-class solutions (all *p* > 0.10). Furthermore, the best-fitting solutions, according to the BIC and ssaBIC values, were the two-class models for all three countries. The entropy values for the two-class models were 0.43, 0.55, and 0.48, in Kenya, Zambia, and Malawi, respectively.

In order to validate the best latent class solution for HIV vulnerability (based on statistical and empirical evidence), the multilevel logistic regression models were used to assess associations between the derived vulnerability classes and different outcome variables (Table 2). The multilevel regression models adjusted for the cluster structure (district level) of the data and age; robust standard errors were produced. All the regression analyses were performed in STATA 13.2 software.

### Ethics and consent

Study protocols were reviewed and approved by the Population Council Institutional Review Board, as well by the Kenyatta National Hospital/University of Nairobi Ethics and Research Committee and National Commission for Science Technology and Innovation in Kenya; College of Medicine Research Ethics Committee at the University of Malawi in Malawi; ERES CONVERGE IRB and the National Health Research Authority in Zambia. Informed consent was obtained from all study participants (or parental consent and respondent assent, as appropriate). As per local ethical research guidelines, participants were compensated for their research participation: KSH300 (approximately US$3) in Kenya, MWK1500 (approximately US$2) in Malawi, and ZMW50 Kwacha (approximately US$5) in Zambia.

## Results

### Sample description

Table 3 presents descriptive characteristics of the study samples from Kenya, Malawi, and Zambia. Mean age of survey respondents was 21 years across all three settings. Approximately 60% of AGYW in Kenya, 48% in Zambia, and 35% in Malawi had lost a parent or both. Marital status of the respondents differed by setting, 64% of the respondents were currently married in Malawi, whereas half were in Kenya and only 19% in Zambia. About 36–40% of the respondents reported being from households with low socio-economic status (SES) across all three settings. Over 20% of the AGYW in Kenya and Malawi had experienced hunger in the past month, whereas 13% in Zambia reported as such. Adult supervision was high, with 60–76% of AGYW reporting that an adult in their household knew where they were during the day or night. Mobility varied by setting, more than two-thirds of respondents in Kenya and Zambia reported being mobile, whereas only 20% of AGYW in Malawi reported traveling outside of their community in the past 12 months. Approximately a third of respondents in each setting had high support for gender equitable norms. In terms of HIV risk, 79% in Kenya, 85% in Zambia, and 53% in Malawi did not think they were at risk of HIV acquisition. About half of the respondents in each setting did not have comprehensive knowledge about HIV, and over 70% in Kenya and Malawi and 49% in Zambia did not have comprehensive knowledge about condoms.

**Table 3:** Descriptive characteristics of study sample, out-of-school 15- to 24-year-old women in Kenya, Malawi, and Zambia, 2016–2017

	Kenya		Malawi		Zambia	
	*N* = 1014		*N* = 1653		*N* = 846	
Average age (SD)	20.6 (2.4)		20.7 (2.3)		20.5 (2.3)	
	*N*	%	*N*	%	*N*	%
Household characteristics						
*Orphanhood*						
Lost both mother and father	230	22.7	130	7.9	108	12.8
Lost mother	285	28.1	328	19.8	227	26.8
Lost father	90	8.9	118	7.1	73	8.6
Not orphan	409	40.3	1077	65.2	438	51.8
*Household socioeconomic status*						
Low	368	36.3	664	40.2	308	36.4
Medium	339	33.4	551	33.3	363	42.9
High	307	30.3	438	26.5	175	20.7
*Hunger in the past month*						
Often/Sometimes	245	24.2	368	22.3	111	13.1
Rarely/never	769	75.8	1285	77.7	735	86.9
*Adult supervision*						
No	302	29.8	243	24.3	324	38.3
Yes	712	70.2	1252	75.7	522	61.7
Respondent characteristics						
*Mobility*						
Weekly/monthly	125	12.3	288	17.4	244	28.8
Yearly	555	54.7	18	1.1	321	37.9
No mobility	334	32.9	1347	81.5	281	33.2
*Marital status*						
Currently married	511	50.4	1049	63.5	159	18.8
Formerly married	54	5.3	263	15.9	50	5.9
Never married	449	44.3	341	20.6	637	75.3
Respondent attitudes						
*Support for gender equitable norms*						
Low	386	38.1	550	33.3	333	39.4
Moderate	329	32.4	551	33.3	285	33.7
High	299	29.5	552	33.4	228	27.0
*Perception on exposure to HIV*						
Somewhat likely/don’t know/high risk	210	20.7	782	47.3	127	15.0
No risk/unlikely	804	79.3	871	52.7	719	85.0
Respondent knowledge						
*Comprehensive knowledge about HIV transmission*						
No	444	43.8	861	52.1	423	50
Yes	570	56.2	792	47.9	423	50
*Comprehensive knowledge about condoms*						
No	746	73.6	1190	72	413	48.8
Yes	268	26.4	463	28	433	51.2

### Model fit statistics

The fit statistics for each model solution are presented in Table 4 with two- and three-latent class solutions. To decide the best-fitting model solution for each country, we used a combination of the BIC, ssaBIC, and LMR *p* value. The two-class solutions demonstrated the most meaningful class interpretation with adequate class sizes and were therefore selected as the best solutions to classify individuals into homogeneous groups for each of the countries.

**Table 4:** Latent class analysis fit indices for two- and three-class solutions among study samples of out-of-school 15- to 24-year-old women in Kenya, Malawi, and Zambia, 2016–2017

Study sample	Class	AIC	BIC	ssaBIC	Entropy	LMR value	*p* value
Kenya	2	16,586.75	16,749.17	16,644.36	0.432	136.47	0.0326
	3	16,563.88	16,809.97	16,651.16	0.509	56.39	0.5653
Malawi	2	24,966.85	25,145.39	25,040.56	0.483	419.78	< 0.0001
	3	24,926.04	25,196.55	25,037.71	0.489	74.23	0.7738
Zambia	2	13,425.29	13,581.73	13,476.93	0.552	111.85	0.0735
	3	13,418.41	13,655.43	13,496.65	0.582	40.54	0.5739

*AIC* Akaike information criterion, *BIC* Bayesian information criterion, *ssaBIC* sample size adjusted, *LMR* Lo–Mendell–Rubin value

### Higher and lower vulnerability profiles among AGYW

Table 5 presents response probabilities by each of the latent classes for each country. We used a cut point of 0.4 to define a response probability as high. We defined class 1 as low HIV vulnerability profile and class 2 as high HIV vulnerability profile.

**Table 5:** Higher and lower vulnerability profiles of out-of-school 15- to 24-year-old women in Kenya, Malawi, and Zambia, 2016–2017

Indicator	Kenya		Malawi		Zambia	
	Class 2	Class 1	Class 1	Class 2	Class 1	Class 2
	Higher vulnerability	Lower vulnerability	Higher vulnerability	Lower vulnerability	Higher vulnerability	Lower vulnerability
Lost both mother and father	0.33	0.17	0.06	0.10	0.13	0.12
Lost mother	0.26	0.29	0.21	0.18	0.27	0.27
Lost father	0.10	0.09	0.08	0.06	0.09	0.08
Low household socioeconomic status	0.27	0.41	0.26	0.56	0.36	0.37
Medium household socioeconomic status	**0.46**	0.27	**0.46**	0.19	0.4	0.46
Hunger in the past month (often/sometimes)	**0.44**	0.14	0.31	0.13	0.16	0.09
No adult supervision	**0.44**	0.22	0.36	0.12	**0.43**	0.32
High mobility (weekly/monthly)	0.19	0.09	0.12	0.24	0.28	0.31
Low mobility (yearly)	0.57	0.54	0.01	0.01	0.31	0.47
Currently married	0.62	0.44	0.69	0.58	0.22	0.14
Formerly married	0.08	0.04	0.17	0.15	0.06	0.06
Lower support for gender equitable norms	**0.68**	0.23	**0.52**	0.13	**0.68**	0.01
Moderate support for gender equitable norms	0.25	0.36	0.35	0.32	0.24	0.47
High perceived risk of exposure to HIV	0.34	0.14	0.51	0.43	0.12	0.19
No comprehensive knowledge of HIV	**0.58**	0.36	**0.76**	0.25	**0.64**	0.31
No comprehensive knowledge of condoms	0.73	0.74	**0.84**	0.59	**0.57**	0.37
Class probability	0.34	0.66	0.53	0.47	0.57	0.43
Classification of individuals (%, *N*)	32.2 (327)	67.8 (687)	53.0 (876)	47.0 (777)	51.5 (436)	48.5 (410)

Bold values denote the key distinguishing characteristic of the latent class profile

In Kenya, the higher HIV vulnerability profile comprised 34% of the sample, whereas the lower vulnerability profile comprised 66% of the sample. Distinguishing characteristics between the two profiles are that the high vulnerability profile had a higher probability of having a medium household SES, being hungry, not having adult supervision, having lower support for gender equitable norms, and having no comprehensive knowledge of HIV. Both the higher and lower vulnerability profile also had equally high probability of low mobility, being currently married, and having no comprehensive knowledge of condoms.

In Malawi, the higher and lower vulnerability profile comprised 53% and 47% of the sample, respectively. Both profiles had high probability of being currently married, high self-perceived risk of HIV exposure, and no comprehensive knowledge of condoms but had low probability of being mobile and being a double orphan. Distinguishing characteristics between the two profiles are that the higher vulnerability profile had high probability of low support for gender equitable norms, medium household SES, and no comprehensive knowledge of HIV.

In Zambia, the higher and lower vulnerability profile comprised 57% and 42% of the sample, respectively. Both profiles had low probability of being currently married, experiencing hunger, and having high self-perceived risk of exposure to HIV, but high probability of having low or medium household SES. Distinguishing characteristics between the two profiles are that the higher vulnerability profile had higher probability of no adult supervision, lower support for gender equitable norms, and no comprehensive knowledge of HIV and condoms.

### Association between HIV vulnerability profiles and HIV status, and key sexual and reproductive health (SRH) outcomes

Table 6 presents multivariate regression results examining associations between AGYW’s HIV vulnerability profiles, HIV status, and key SRH outcomes, after adjusting for site and age. In all three settings, being in the higher vulnerability class compared to the lower vulnerability class was associated with a range for poor SRH and HIV outcomes among AGYW. In Kenya, AGYW who were in the higher vulnerability class were at significantly greater risk of already living with HIV and had significantly greater odds of having experienced STI symptoms in the past six months, a very early sexual debut, a pregnancy, multiple sexual partners in the past year, physical and sexual violence from intimate partners, and sexual violence from non-intimate partners. Being in the high vulnerability class was also marginally associated with engaging in transactional sex.

**Table 6:** Multivariate associations between HIV vulnerability profiles and HIV status, and sexual and reproductive health outcomes among out-of-school 15- to 24-year-old women, Kenya, Malawi, and Zambia, 2016–2017

	Kenya (*N* = 1014)			Malawi (*N* = 1653)			Zambia (*N* = 846)		
	HIV vulnerability profile			HIV vulnerability profile			HIV vulnerability profile		
	Low HIV vulnerability	High HIV vulnerability	*p* value	Low HIV vulnerability	High HIV vulnerability	*p* value	Low HIV vulnerability	High HIV vulnerability	*p* value
HIV positive (%)	3.8	11.3		1.3	2.1		2.7	3.2	
AOR (95% CI)	Ref	2.75 (1.62–4.67)	< 0.001	Ref	1.61 (0.74–3.51)	0.230	Ref	1.25 (0.56–2.79)	0.586
Sexually transmitted infection symptoms (%)	18.8	26.9		26.4	34.4		14.9	14.9	
AOR (95% CI)	Ref	1.57 (1.14–2.15)	0.005	Ref	1.44 (1.16–1.78)	0.001	Ref	1.00 (0.68–1.47)	0.99
First sex ≤ 15 years (%) (Kenya = 896; Malawi = 1569; Zambia = 626)	37.4	47.7		30.1	34.9		11.4	22.3	
AOR (95% CI)	Ref	1.73 (1.29–2.31)	< 0.001	Ref	1.24 (1.00–1.54)	0.046	Ref	2.15 (1.37–3.37)	0.001
Pregnancy experience (%)	67	89.9		82.5	90.4		40.2	52.1	
AOR (95% CI)	Ref	3.71 (2.45–5.61)	< 0.001	Ref	2.19 (1.63–2.95)	< 0.001	Ref	2.03 (1.50–2.74)	< 0.001
Engaged in transactional sex (%) (Kenya = 930; Malawi = 1569; Zambia = 628)	5.2	7.9		2.5	4.4		3.4	8.6	
AOR (95% CI)	Ref	1.72 (0.99–2.97)	0.054	Ref	1.74 (0.98–3.10)	0.058	Ref	2.66 (1.27–5.57)	0.009
Multiple sexual partners (%) (Kenya = 675; Malawi = 1192; Zambia = 360)	11.6	24.7		15.4	17.4		4.9	8.7	
AOR (95% CI)	Ref	2.75 (1.79–4.22)	< 0.001	Ref	1.16 (0.84–1.60)	0.357	Ref	1.84 (0.77–4.38)	0.17
HIV test in the past 12 months (%)	94.8	96		93.1	96.5		70	70.4	
AOR (95% CI)	Ref	1.16 (0.60–2.24)	0.661	Ref	1.04 (0.94–1.14)	0.484	Ref	1.10 (0.81–1.48)	0.555
Condom use at last sex (%) (Kenya = 675; Malawi = 1439; Zambia = 324)	34	30.5		16.9	18.6		43.3	27	
AOR (95% CI)	Ref	0.91 (0.64–1.29)	0.6	Ref	1.11 (0.84–1.46)	0.448	Ref	0.45 (0.28–0.72)	0.001
Experienced sexual violence from intimate partners (%) (Kenya = 684; Malawi = 1561; Zambia = 654)	12.8	29.5		16.5	19		18.5	30.3	
AOR (95% CI)	Ref	2.79 (1.87–4.17)	< 0.001	Ref	1.12 (0.86–1.46)	0.417		1.90 (1.32–2.74)	0.001
Experienced of physical violence from intimate partners (%) (Kenya = 684; Malawi = 1561; Zambia = 654)	23.8	43.3		14.7	17.3		21.8	37.3	
AOR (95% CI)	Ref	2.31 (1.62–3.30)	< 0.001	Ref	1.26 (0.95–1.66)	0.1		2.21 (1.56–3.12)	< 0.001
Experienced sexual violence from non-intimate partners (%)	16.9	26.9		7.9	10.4		17.6	15.1	
AOR (95% CI)	Ref	1.94 (1.40–2.69)	< 0.001	Ref	1.36 (0.95–1.92)	0.087		0.83 (0.57–1.20)	0.315

*AOR* Adjusted odds ratios. All regression estimates are based on multilevel model and adjusted for the cluster structure (district) and age

In Malawi, being in the higher vulnerability class was associated with increased odds of having experienced STI symptoms, very early sexual debut, and pregnancy. Being in the high vulnerability class was also marginally associated with engaging in transactional sex, and experience of physical violence from intimate partners and sexual violence from non-partners.

In Zambia, AGYW in the higher vulnerability class had increased risk of engaging in transactional sex and increased odds of very early sexual debut, having a pregnancy experience, experiencing physical and sexual violence from intimate partners, and had lower odds of condom use at last sex. There were no significant associations with recent HIV testing and vulnerability profiles in any setting.

## Discussion

We use quantitative data gathered with over 3000 AGYW from three country contexts tapping individual- and household-level factors to identify distinct profiles of HIV vulnerability among AGYW. This multi-country analysis shows that even in communities with high HIV prevalence, not all AGYW are equally at risk of HIV. We find two distinct profiles of HIV vulnerability (higher and lower) among out-of-school AGYW in Kenya, Malawi, and Zambia. Using less-sensitive questions, which measure individual and social factors more distally associated with HIV risk, this analysis identifies profiles of AGYW who are more vulnerable to HIV in high HIV prevalence settings. This analysis contributes knowledge toward the key challenge of identifying and reaching vulnerable AGYW for HIV prevention efforts, as being in the higher vulnerability profile was associated with a range of poor SRH outcomes that are proximally related to HIV acquisition. This segmenting approach provides a more nuanced understanding of factors that synergistically constitute higher vulnerability for out-of-school AGYW in each country context, and it offers the possibility for initiating a conversation about risk and could potentially lead to the development of more effective targeting of subgroups of young women who need to be urgently reached with HIV prevention programming.

In our analysis, we find that some factors that define vulnerability are context specific, while others consistently contribute to HIV vulnerability across settings. In urban/peri-urban Kenya for instance, being hungry/food insecurity—which has been linked with increased sexual risk and inability to negotiate safe sexual practices (Chop et al. 2017)—contributed to the higher HIV vulnerability profile, whereas this was not the case in Malawi or Zambia. In our analysis, parental supervision was also a contributing factor for vulnerability in urban/peri-urban Kenya and urban Zambia. Recent research in Kenya and the region has shown that family connectedness and parental monitoring were associated with less sexual risk taking (Cluver et al. 2016; Wachira et al. 2019). In both Kenya and Malawi, coming from a household that did not have the poorest or the richest SES relative to the community was also a key factor in vulnerability. In the fairly impoverished communities in our study, it could mean that a medium socioeconomic status household may not have access to social support structures available to the poorest households. Prior research in Malawi has also shown a similar relationship between household SES and HIV and found that young people living in comparatively better off households/areas are more likely to have HIV, likely due to their proximity to roads and mobile populations (Mensch and Soler-Hampejsek 2017). Across the three country contexts, having gender inequitable attitudes and no comprehensive knowledge about HIV were consistently associated with the higher vulnerability profile. The prominent influence of these factors in defining the higher vulnerability profile across all three contexts may mean that these may be essential elements for HIV prevention programming to focus on/consider. Prior research has demonstrated the link between inequitable gender norms and sexual risk behaviors (Gottert et al. 2018). Recent research among AGYW in the region has shown that having inequitable gender attitudes had the highest predicted probability of HIV acquisition (Wesson et al. 2019). Similarly, comprehensive sexuality education, particularly programs that take an empowerment approach and discuss gender and power, has been shown to be associated with lower risk-taking behaviors (Boonstra 2015; Haberland and Rogow 2015). The persistence of these two factors in defining the higher vulnerability profile signals the need for greater attention to these factors collectively in intervention design and messaging. For instance, ensuring comprehensive knowledge about HIV transmission and condoms among AGYW, and addressing gender roles and norms at the individual and community levels could shift HIV vulnerability for AGYW.

Our study uses the segmentation approach (Sgaier et al. 2018) to identify HIV vulnerability profiles for AGYW living in sub-Saharan Africa. While segmentation approaches have long been used in business and marketing fields, their application in global public health efforts remains limited (Gomez et al. 2018). Notable exceptions include a study in Malawi that examined HIV risk perception and self-efficacy dimensions to better inform the development of tailored HIV prevention messages to different subgroups of men and women (Rimal et al. 2009), work in Zambia and Zimbabwe on understanding the underlying drivers for men’s decisions for voluntary medical male circumcision, and work in Niger around developing profiles of women’s willingness to adopt family planning (Camber Collective 2015; Dalglish et al. 2018). To-date, segmentation using LCA has been used only sparingly in HIV research in sub-Saharan Africa, a region with the highest HIV burden, and where this approach may offer critical insights around HIV vulnerability and improved targeting. This analysis is an initial attempt at the application of such an approach across three countries and adds new knowledge to this burgeoning field.

We conducted this examination to assess whether non-sensitive questions could be used to define HIV vulnerability profiles among out-of-school AGYW. The LCA allowed us to look at the set of factors that together/synergistically constitute higher vulnerability in each country context, and regression analyses confirmed that AGYW in the higher vulnerability profile in each country were more likely to be engaging in a range of risky behaviors, and subject to sexual violence compared to AGYW in the lower vulnerability profile—putting them at greater risk of HIV acquisition. A next step would be to translate these findings for use in HIV prevention program screening tools. Since our analysis used a set of non-sensitive measures to assess HIV vulnerability, these measures could be captured and used at the community level. Some of the items used in our analysis (e.g., GEM scale) would need to be pared down for ease of use and interpretation. This type of segmentation analysis could also lead to better programmatic evaluations. Subsequent work can assess whether AGYW in the higher vulnerability profiles have increased exposure and uptake of program interventions, whether there is a shift in the vulnerability profile, and whether there are changes in risk behaviors among AGYW in the high vulnerability group. From a policy perspective, we hope this study highlights for the global health and development sector practitioners and donors the need for more effective segmentation or profiling of target audiences for intervention design and implementation.

Our study has some limitations. Self-reported data from AGYW may be subject to bias. AGYW may have underreported/may not have disclosed their HIV status. Our study inclusion criteria (e.g., intention to stay in the area) could also be introducing bias. This analysis also relies on cross-sectional data, and therefore outcomes, such as sexual violence, are reported retrospectively. This analysis presents an initial step in applying a segmentation approach for outreach in HIV prevention programming but may have applicability in other public health sectors as well. Future work should assess whether these profiles and this approach is useful for targeting highly vulnerable girls in HIV prevention programming and evaluation efforts. Subsequent examinations could consider stratifications by age or marital status for refinement of vulnerability profiles. These profiles could be considered in similar contexts within Kenya, Malawi, and Zambia among out-of-school AGYW. Additional work is needed to examine whether these findings could be generalized to other contexts or to develop context-specific vulnerability profiles.

### Conclusion

To stem the HIV epidemic, HIV prevention programs need to reach the right people with the right interventions. It is often a challenge to identify whom to reach in high HIV prevalence settings and to identify HIV vulnerability. We found two distinct profiles of risk among out-of-school AGYW, defined by a grouping of factors that synergistically influence HIV vulnerability. Our analysis found that AGYW in the higher vulnerability class had increased odds of negative health outcomes and experiences, confirming that the higher and lower vulnerability profiles are distinct. These analyses provide insights on the need to tailor community-based HIV prevention efforts by differentially targeting/tailoring interventions and health services for subpopulations in higher versus lower HIV vulnerability profiles.
